# A Novel Green Synthesis Method of Copper Nanoparticles and Their Biological Effects on Cancer and Normal Cells

**DOI:** 10.3390/ijms27062559

**Published:** 2026-03-11

**Authors:** Maria-Alexandra Pricop, Adina Negrea, Ioan Bogdan Pascu, Mihaela Ciopec, Petru Negrea, Iustina-Mirabela Cristea, Călin Adrian Tatu, Alexandra Ivan

**Affiliations:** 1 OncoGen Centre, County Hospital Pius Branzeu, 156 Liviu Rebreanu Blvd, 300723 Timisoara, Romania; alexandra.pricop@oncogen.ro (M.-A.P.); mirabela.cristea@oncogen.ro (I.-M.C.); ivan.alexandra@umft.ro (A.I.); 2 Department of Applied Chemistry and Environmental Engineering and Inorganic Compounds, Faculty of Industrial Chemistry, Biotechnology and Environmental Engineering, Politehnica University Timisoara, Vasile Pârvan 6, 300223 Timisoara, Romania; adina.negrea@upt.ro (A.N.); mihaela.ciopec@upt.ro (M.C.); petru.negrea@upt.ro (P.N.); 3 Renewable Energy Research Institute-ICER, Politehnica University of Timisoara, 138 Gavril Musicescu Street, 300501 Timisoara, Romania; ioan.pascu@upt.ro; 4 Department of Functional Sciences, University of Medicine and Pharmacy “Victor Babes”, Eftimie Murgu Sq. 2, 300041 Timisoara, Romania

**Keywords:** CuNPs, nanoparticles, starch, in vitro cytotoxicity, oxidative stress, apoptosis

## Abstract

Copper-based nanoparticles (Cu-based NPs) represent a major focus in nanomedicine due to their unique physicochemical properties and excellent biocompatibility. In this paper, we present an interdisciplinary study bridging engineering and biomedical sciences by employing a novel synthesis approach to produce highly stable and uniformly dispersed spherical copper nanoparticles (CuNPs), which were subsequently tested for their cytotoxic effects on SKBR3 and MSC human cells. The synthesis of CuNPs was performed in the presence of the complexing agent trisodium citrate (TSC), while starch was used for the chemical reduction step. Characterization of the Cu-based NPs via UV–Vis, FT-IR, Mie theory, DLS and SEM confirmed their nanoscale structure. The obtained CuNPs were subsequently assessed for their biological effects and cytotoxic responses induced in normal and SKBR3 cancer cell lines. The SKBR3 cell line showed a dose-dependent decrease in the cell index and a higher proportion of apoptotic cells compared to normal MSCs, with apoptosis representing the dominant mode of cell death. Although SKBR3 cells appeared to mount an antioxidant response against CuNP oxidative stress, the response was insufficient to counteract the apoptotic progression. In comparison, MSCs showed a greater resilience to CuNP-induced cellular stress. By promoting oxidative stress and disrupting the antioxidant defense system of cancer cells, CuNPs exhibit promising anti-cancer properties.

## 1. Introduction

Nanotechnology has become a central and transformative discipline in modern science, the bridging engineering and medical fields. The main purpose of nanoscience is the production of materials on a nanometric scale with novel physical, chemical, and biological properties that can lead to innovative applications in fields like medicine, electronics, and engineering [1,2]. Among these nanomaterials, copper nanoparticles (CuNPs) have attracted considerable attention due to their distinctive physicochemical properties and broad range of potential applications, especially in medicine, where they offer innovative strategies for disease diagnosis and therapy [3,4,5,6,7,8,9].

Technological advances indicate that copper-based nanoparticles (Cu-based NPs) are expected to play an increasingly important role in addressing some major societal challenges, from the development of sustainable technologies to improvement of health outcomes [1]. Furthermore, advanced synthesis strategies—particularly chemical reduction methods—are paramount as they grant precise control over the size, shape, and surface chemistry of CuNPs. Although highly efficient, chemical reduction methods often pose challenges related to excessive reaction rates, toxicity, and poor environmental compatibility, which can ultimately affect nanoparticle stability and dispersion [10,11,12]. In recent years, natural polymers have been increasingly used in nanoparticle synthesis following green chemistry principles. By comparison, starch offers a sustainable and environmentally friendly alternative, combining mild reducing ability with high availability, low cost, and excellent solubility in aqueous systems. Upon thermal activation, its polymeric structure enables the gradual release of reduced saccharide units, allowing better control over reduction kinetics and nanoparticle growth. The hydroxyl groups in starch can contribute to the reduction in metal ions and also act as stabilizing agents, preventing nanoparticle aggregation [13]. Several studies have reported the successful use of starch in the synthesis of metal nanoparticles, supporting its role as an eco-friendly alternative to conventional materials. Suramwar et al. (2016) reported that cornstarch enabled one-pot synthesis of metallic CuNPs at room temperature [13]. Alishah et al. (2017) employed potato starch in the green synthesis of CuO nanoparticles, where it facilitated partial reduction and stabilization [14]. Rice starch has also played a role in generating CuO nanocomposites through ultrasonic methods [14]. More recently, Chen (2022) highlighted starch’s dual role as a mild reducing agent and an encapsulating matrix for CuO nanostructures [15]. However, the relatively weak reducing capacity of starch often results in copper oxides rather than pure copper, while also posing challenges in achieving precise control over particle sizes and long-term stability [16].

Starch-mediated copper nanostructures have also been investigated for biomedical applications, showing promising applicability in cancer treatment. Alishah et al. (2017) reported a significant reduction in the viability of MCF-7 breast cancer cells by more than 60% at 100 µg/mL, Ilbasmis-Tamer et al. (2023) observed pronounced apoptotic and necrotic responses in Capan-1 pancreatic cancer cells, and Hou et al. (2022) demonstrated strong anti-proliferative effects of Fe_3_O_4_@starch/Cu nanocomposites against ovarian cancer models, highlighting their promising cytotoxic activity across different tumor types [14,17,18]. Although copper is traditionally known for its direct cytotoxic effects—primarily mediated through oxidative stress induction—its role in immunotherapy is considerably more complex and context-dependent. CuNPs in cancer immunotherapy represent an emerging and rapidly advancing field that combines the distinct physicochemical properties of copper with the immune system’s ability to recognize and eliminate cancer cells. In this context, CuNPs can promote immunogenically induced cell death, a regulated form of cell death characterized by the releases of danger-associated molecules. These molecular, alarm signals enhance tumor immunogenicity, effectively converting an “invisible” tumor into a target that can be actively recognized and destroyed by the patient’s immune system [19,20,21]. However, despite ongoing research, a significant challenge persists: CuNPs often exhibit cytotoxicity toward human cells compared to other metal oxide nanoparticles. In vitro studies have showed that CuNPs can induce multiple toxic effects, including membrane injury and increased oxidative stress, ultimately leading to apoptosis [22]. To fully understand the mechanisms of CuNPs, improve their therapeutic potential, and ensure their safety and efficacy in clinical settings, further research is necessary, especially since copper complexes have a relatively shorter history in cancer therapy compared to other metal complexes. Therefore, any newly developed copper-based material with anti-cancer properties must undergo comprehensive evaluation before translating into medical applications.

**Figure 1:**
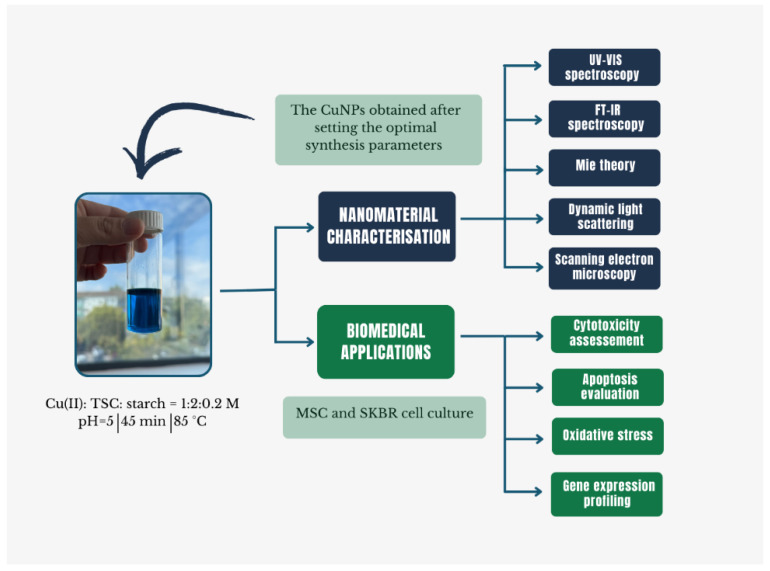
Synoptic overview of optimal synthesis conditions and biological evaluations of CuNPs.

The primary objective of this work was the synthesis of CuNPs and the systematic investigation of the chemical parameters that influence the nanomaterial formation via a chemical reduction route. The CuNPs obtained via a green novel method, TSC, were initially used to complex Cu(II) ions, while starch served as a dual-function component, acting both as a reducing agent and stabilizer. The second objective of the study was to evaluate starch as a green reducing and stabilizing agent within the synthesis process. The green synthesis approach of CuNPs offers an alternative to the conventional chemical reduction methods that rely on strong reducing agents such as sodium borohydride, hydrazine, ascorbic acid, or polyols to achieve rapid reduction in Cu(II) ions [12,23,24]. Although starch is not a traditional reducing agent, it represents an environmental friendly approach due to its biodegradability, low cost, and biocompatibility, while securing colloidal stabilization by increasing solution viscosity and providing steric hindrance, eliminating the need for hazardous stabilizing agents [25,26,27,28]. Furthermore, the biological effects of the synthetized CuNPs were evaluated by assessing cytotoxicity, apoptosis, intracellular ROS levels and the expression of oxidative stress-related genes (CAT, SOD and PPARγ) in both normal and cancer cells. A schematic overview of the experimental design is presented in Figure 1.

## 2. Results

### 2.1. Optimization of Synthesis Parameters for Copper Nanoparticles via Chemical Reduction

#### 2.1.1. The Role of TSC as a Complexing Agent in the CuNP Synthesis

**Figure 2:**
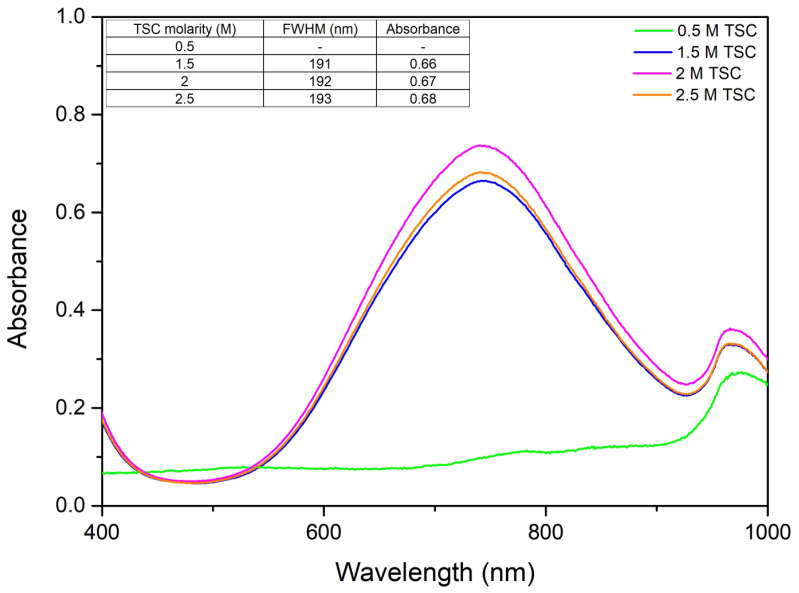
TSC variation effect on the UV–Vis spectra.

The choice of complexing agent in nanoparticle synthesis is critical as it influences particle size, shape, stability, and dispersion. By varying the amount of TSC and keeping the amounts of precursor and reducing agent constant, the following UV–Vis spectra were obtained (Figure 2).

According to Figure 2, the surface plasmon resonance existing at wavelength λ = ~746 nm indicates the presence of CuNPs [29]. It can be observed from the resulting spectra that with an increase in TSC concentration the absorbance increases, thus indicating an increase in CuNPs present in the solution. At a higher concentration of TSC there is a decrease in absorbance, indicating a decrease in CuNP production. This can be due to the fact that a higher concentration of TSC may lead to further coalescence of the nanomaterial, developing coarser CuNPs. In order to study how TSC concentration affects monodispersity, the full width half maxima (FWHM) was determined. From here we can see that the monodispersity of the colloidal system is not affected so much with the increase in TSC.

#### 2.1.2. The Role of the Reducing Agent, Starch, in the CuNP Synthesis

**Figure 3:**
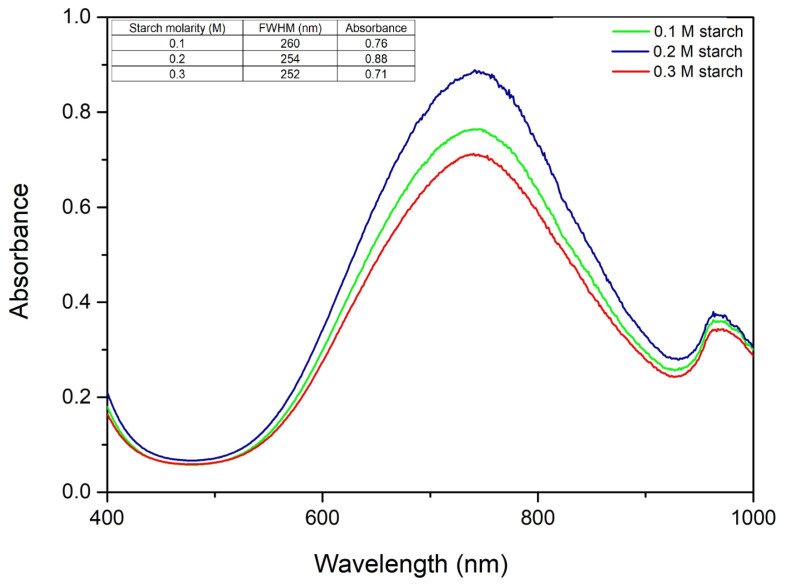
Starch concentration variation and its effect on the UV–Vis spectra.

The chemical reduction method utilizing starch is widely adopted due to its dual role as a natural reducing and stabilizing agent, which facilitates mild reaction conditions and enhances control over nanoparticle size. Precise optimization of starch concentration during CuNP synthesis is critical in obtaining nanoparticles with targeted size, morphology, and physicochemical properties. The rate at which copper ions are reduced to metallic copper is directly influenced by the concentration of the starch. An excessive amount might cause precipitation of the solution. Figure 3 shows the UV–Vis spectra for the molar ratios of Cu(II):TSC:starch, varying the amount of starch and keeping the amounts of precursor and complexing agent constant.

From the resulting spectra, it can be observed that the molar ratio of Cu(II):TSC:starch = 1:2:0.2 gives the highest absorbance, which is correlated with the highest concentration of CuNPs. The full width at half maximum and the absorbance values are also presented in Figure 3. From the results obtained, it is observed that the values of full width at half maximum are relatively similar, indicating that there are no major differences in terms of the monodispersity of the colloidal system. However, there are differences in terms of absorbance, the optimal one being 254 nm, which belongs to the molar ratio Cu(II):TSC:starch = 1:2:0.2, considered the optimal synthesis ratio.

The obtained spectra demonstrate that absorbance increases proportionally with the starch content, indicating a higher concentration of synthesized copper nanoparticles [30], until the starch concentration reaches 0.3 M, at which point a decline in absorbance is observed, suggesting a decrease in the concentration of formed nanoparticles [30]. This phenomenon may be attributed to the fact that an excess of starch induces the coalescence of particles, resulting in the formation of larger copper nanoparticles and a subsequent decrease in the SPR intensity. FWHM was also determined in order to observe how starch concentration affects monodispersity. From what we have uncovered, we can observe that at 0.2 M starch FWHM is smaller, indicating a higher colloidal monodispersity, and even though at a higher concentration of starch FWHM drops, since the absorbance drops considerately, we can conclude that the optimal starch concentration is 0.2 M, thus further studies were carried out with 0.2 M starch concentration.

#### 2.1.3. The Role of pH in the CuNP Synthesis

The pH acts as a key parameter that regulates the expansion of starch chains, directly governing the particles’ final size and morphology. To evaluate the influence of medium alkalinity on the synthesis, the pH was varied while keeping the concentrations of starch and TSC constant, at the optimal ratios determined in the previous steps.

**Figure 4:**
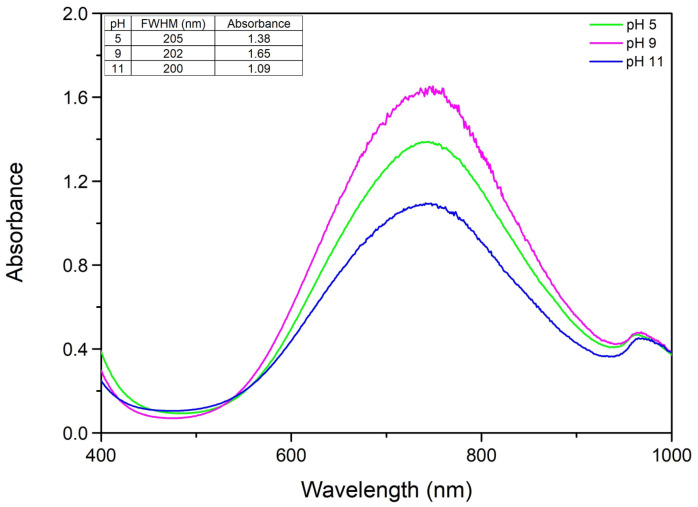
The role of pH variation on the UV–Vis spectra.

From the observed spectra (Figure 4) we can conclude that in this situation a more acidic pH, pH 5, is the best option for this type of synthesis, unlike conventional nanoparticle synthesis where a more alkaline pH is usually more suitable [31,32]. Even though at pH 9 our CuNPs have a bigger absorbance, the fact that we got too much noise implies that the colloidal system is changing and this can be due to morphology changes in the particles. We can observe that at a higher pH, pH 11, the absorbance is smaller. This can be related to the fact that a higher pH may cause copper to form copper hydroxide (Cu(OH)_2_) which will lead to the formation of coarse Cu(OH)_2_ particles, which in time will settle at the bottom of the glass. We have also determined the FWHM in order to observe how pH affects monodispersity and from the results we can conclude that the pH in this type of synthesis does not play a major role in the colloidal monodispersity. The conclusion of this experiment is that the optimal pH is 5.

#### 2.1.4. The Role of Homogenization Time in the CuNP Synthesis

**Figure 5:**
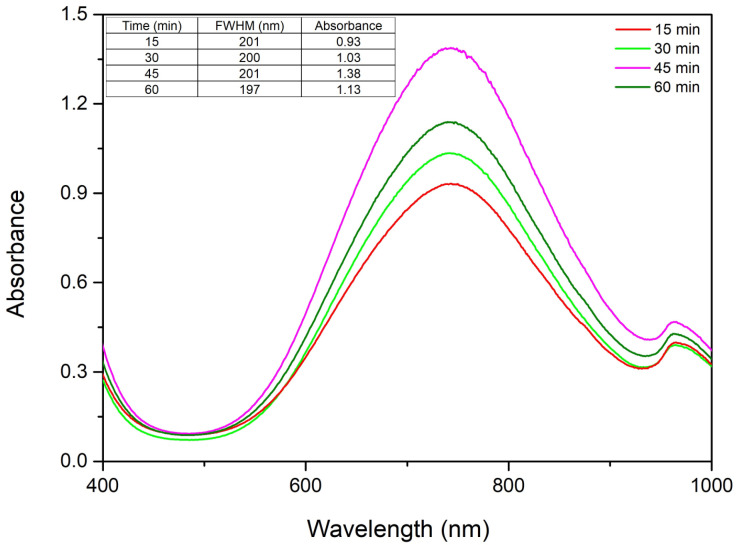
The role of homogenization time on the UV–Vis spectra.

The reaction time during the synthesis of copper nanoparticles using starch as a reducing and stabilizing agent is a critical parameter. From the obtained results, in Figure 5 it is observed that with the increase in the homogenization time, the absorbance increases and implicitly the CuNP concentration increases, which indicates that the monodispersity of the colloidal system increases [33]. As no major changes in absorbance or full width at half maximum occur between 60 and 90 min, a homogenization time of 45 min was considered sufficient for subsequent studies.

From the UV–Vis spectra we can observe that a higher stirring time facilitates the synthesis of CuNPs, until we reach 45 min, due to the increase in absorbance with the increase in homogenization time. When we reach 60 min of homogenization time the absorbance drops, indicating a decrease in CuNP concentration, thus the optimal stirring time is 45 min. In order to fully understand the effect of reaction time on the colloidal monodispersity, FWHM was determined and we can observe that there is no major modification.

#### 2.1.5. The Role of Temperature in the CuNP Synthesis

**Figure 6:**
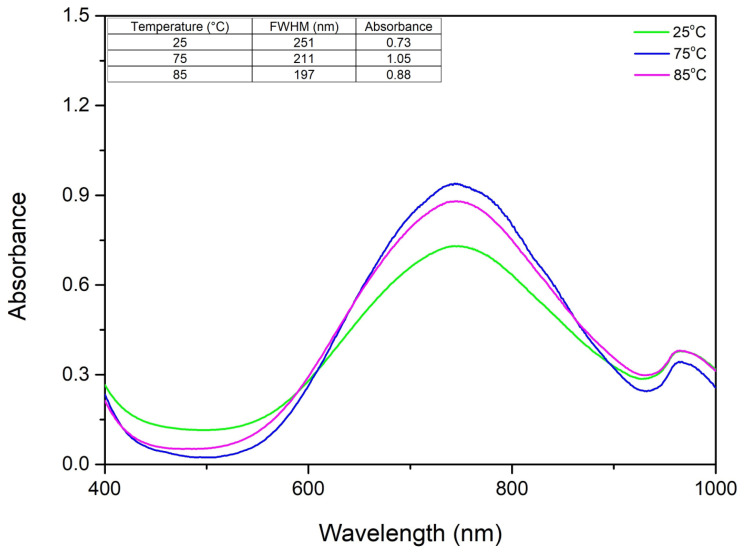
The role of temperature on the UV–Vis spectra.

Temperature is a key factor affecting particle size. The samples were evaluated at temperatures between 25 and 85 °C, maintaining an unchanged molar ratio of Cu(II):TSC:starch = 1:2:0.2, pH = 5 and homogenization time of 45 min. In Figure 6, the UV–Vis spectrum results suggest that the optimal temperature for the synthesis is 85 °C.

It was revealed that an increase in temperature correlates with a rise in absorbance, indicating a higher concentration of synthesized copper nanoparticles. This is likely because higher temperatures cause starch molecules to absorb water and burst, releasing the glucose needed to drive the synthesis of the nanoparticles. To better understand how temperature affects the colloidal system, we determined the FWHM and observed a sharp drop as the temperature increased. While this decrease becomes less significant above 75 °C, it continues to trend downward. We concluded that 85 °C is the optimal temperature because it produces a clear UV–Vis spectrum with no background noise and the narrowest FWHM, indicating a stable and consistent synthesis.

### 2.2. The Mechanism of Copper Nanoparticle Synthesis

Green synthesis of copper nanoparticles follows a controlled chemical reduction mechanism involving distinct nucleation and growth stages, governed by a molar ratio of 1:2:0.2 between the CuNPs precursor, trisodium citrate (TSC), and soluble starch. The process begins with the formation of a stable copper–citrate complex, which regulates the release of free Cu(II) ions and prevents premature precipitation under alkaline conditions [34]. With the addition of pre-activated starch at pH 13, the biopolymer undergoes partial alkaline hydrolysis and deprotonation, leading to the fragmentation of amylopectin and amylose chains into smaller oligosaccharide units with enhanced reducing capacity [35].

When the system is heated to 85 °C, aldehydic groups released from these saccharide fragments are oxidized to carboxylate species, supplying the electrons required for the reduction in Cu^2+^ to Cu^+^. Simultaneously, long-chain starch molecules act as a capping agent, providing steric stabilization that inhibits nanoparticle aggregation and protects the metallic core against oxidation. As a result, a stable and monodisperse colloidal suspension is obtained [36].

In the first stage, an aqueous 1 M Cu(II) solution is treated with trisodium citrate at a molar ratio of 1:2 (Cu^2+^:TSC). Trisodium citrate dissociates to generate citrate ions $\left[C_{6}H_{5}O_{7}{]}^{3-}\right.$, which act as multidentate ligands and readily chelate Cu^2+^ ions. This chelation leads to the formation of stable copper–citrate complexes, significantly decreasing the concentration of free Cu^2+^ species in solution and regulating their chemical availability. Citrate ions complex Cu^2+^ in a 1:1 molar ratio, as described by the reactions reported in the literature as follows [33]:

(1) $$2{Cu}^{2+}\;+\;2{\left[C_{6}H_{5}O_{7}\right]}^{3-}⇌\;{\left[{Cu}_{2}{\left(C_{6}H_{5}O_{7}\right)}_{2}\right]}^{2-}$$

where the stability constant log β = 14.43.

Separately, starch with the general formula $\left(C_{6}H_{10}O_{5}{)}_{n}\right.$ is dispersed in an alkaline medium and adjusted to pH 13 prior to its addition to the reaction system. Under strong alkaline conditions, starch undergoes partial deprotonation of hydroxyl groups and limited alkaline hydrolysis, resulting in increased solubility and the formation of saccharide fragments with reducing properties [37]. This alkaline activation enhances the reducing capability of starch and facilitates its subsequent participation in the redox process. The proposed starch activation reaction is presented below:

(2) $$\left(C_{6}H_{10}O_{5}{)}_{n}\;+\;{\mathrm{nOH}}^{-}\to \;\mathrm{alkaline-activated}\;\mathrm{starch}\;+\;\mathrm{reducing}\;\mathrm{oligosaccharides}\right.$$

Soluble starch is mainly composed of amylose (10–30%) and amylopectin (70–90%). With increasing pH, amylopectin chains undergo cleavage into glucose fragments, which possess the ability to reduce Cu(II) ions [30]. Upon heating the reaction mixture to 85 °C, the reducing saccharide units generated from starch become chemically active [37]. The aldehydic groups present in glucose and oligosaccharide fragments are oxidized to carboxylate species, supplying the electrons required for the reduction in Cu(II).

(3) $$\mathrm{R-CHO}+3O{H\;}^{-}\to {\mathrm{R-COO}}^{-}+2H_{2}O+2e^{-}$$

The electrons generated during the oxidation process are transferred to Cu(II) ions coordinated within the citrate complex, thereby driving the reduction reaction as follows:

The specific copper reduction reaction:

(4) $$2{\mathrm{Cu}}^{2+}\;+\;2HO^{-}+\;2e^{-}\to {{\mathrm{Cu}}_{2}O}_{\left(s\right)}\;+\;H_{2}O\;$$

Global reaction:

(5) $$C_{6}H_{12}O_{6}\;+\;[{{\mathrm{Cu}}_{2}(C_{6}H_{5}O_{7}{)}_{2}]}^{2-}\;+\;5O{H\;}^{-}\to {C_{6}H_{11}O_{7}}^{-}+{Cu}_{2}O_{\left(s\right)}+2{C_{6}H_{5}O7}^{3-}+3H_{2}O$$

### 2.3. Characterization of Synthesized CuNPs

#### 2.3.1. FT-IR Analyses

**Figure 7:**
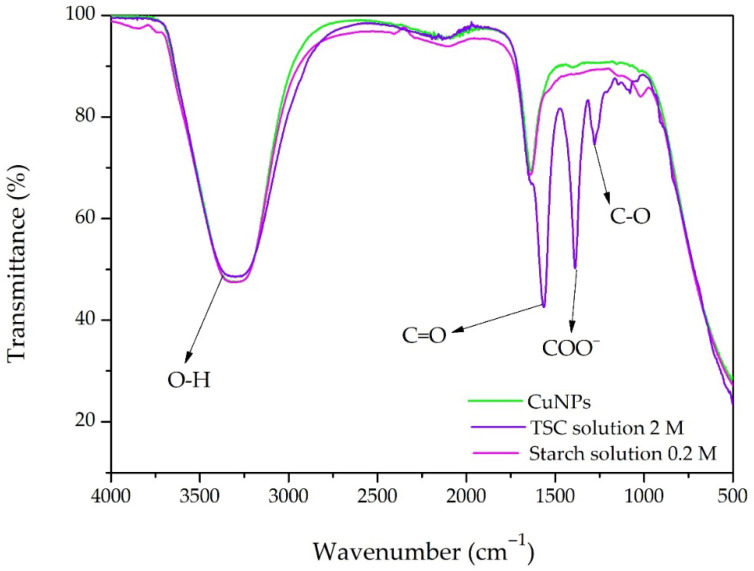
FT-IR spectral analysis of CuNP synthesis and the chemical reagents (TSC and starch) involved in the reaction.

To highlight the functional groups of the synthesized CuNPs, an FT-IR investigation was performed, as shown in Figure 7.

The wide band in the range of 3200–3500 cm^−1^ is assigned to O–H stretching vibrations due to hydroxyl groups of starch, citrate molecules, and adsorbed water [38]. The band in the range of 1650–1720 cm^−1^ is due to the C=O stretching vibration of citrate carbonyl groups. Moreover, the strong peaks in the range of 1550–1600 cm^−1^ and 1350–1450 cm^−1^ are assigned to the asymmetric and symmetric stretching modes of carboxylate (COO^−^) groups, respectively. In addition, the absorption band at 1277 cm^−1^ is assigned to C–O stretching vibrations of carboxylate. Overall, these spectral changes indicate the interaction of starch and trisodium citrate functional groups with CuNPs, and thus demonstrate their dual role as reducing and stabilizing agents via coordination with hydroxyl and carboxylate groups [38].

#### 2.3.2. Mie Scattering Theory

**Figure 8:**
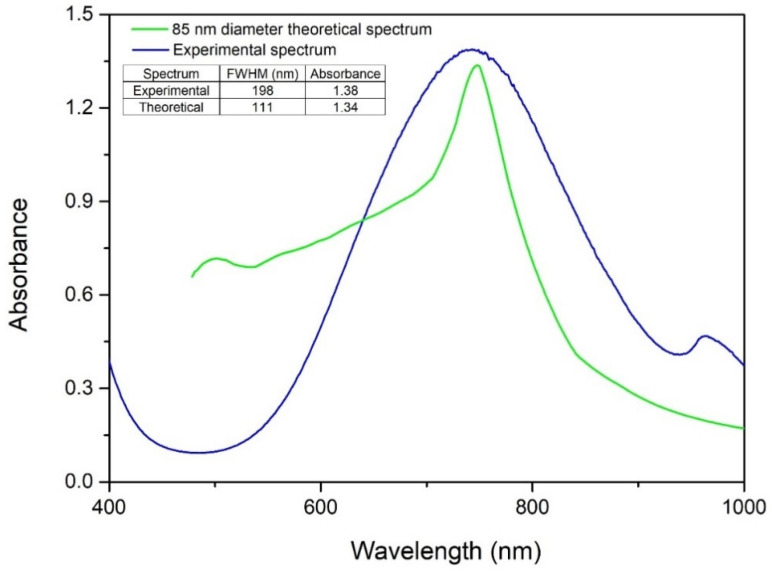
Theoretical size assessment using Mie theory.

To determine the particle size, Mie theory was employed to simulate a theoretical spectrum for comparison with experimental data as shown in Figure 8.

The simulation corresponding to a nanoparticle diameter of 85 nm exhibited a localized surface plasmon resonance (LSPR) at a wavelength closely matching the experimental peak, indicating an average particle diameter of approximately 85 nm. In addition, the similarity in absorbance intensities suggests comparable nanoparticle concentrations in both the simulated and experimental systems. A noticeable discrepancy was observed between the theoretical full width at half maximum (FWHM) of 111 nm and the experimentally measured FWHM of 198 nm. This difference can be attributed to the idealized assumption of perfect monodispersity in the Mie simulations, whereas the synthesized CuNPs exhibit an inherently polydisperse size distribution. Despite this difference in spectral broadening, the close alignment of the LSPR peak positions supports the conclusion that the average diameter of the synthesized nanoparticles is approximately 85 nm.

#### 2.3.3. Dynamic Light Scattering (DLS)

The experimental data indicate a well-controlled particle size and a highly uniform surface charge distribution for the synthesized copper nanoparticles (CuNPs). Dynamic Light Scattering (DLS) analysis of the colloidal suspension revealed an average hydrodynamic diameter of 76.7 nm.

#### 2.3.4. Zeta Potential Measurement

A pronounced mean zeta potential of −57.4 mV was measured, corresponding to an average electrophoretic mobility of −4.47 × 10^−4^ cm^2^ V^−1^ s^−1^. The intensity-weighted size distribution exhibits a single, narrow, and symmetric peak, with no secondary populations detected, confirming the monodisperse nature of the system with respect to the nanoparticle surface charge. The high negative zeta potential indicates strong electrostatic repulsion between particles, which significantly enhances colloidal stability [39]. In nanotechnology, values exceeding ±30 mV indicate strong electrostatic repulsion sufficient to overcome van der Waals attraction [40].

#### 2.3.5. Scanning Electron Microscopy Analysis (SEM)

**Figure 9:**
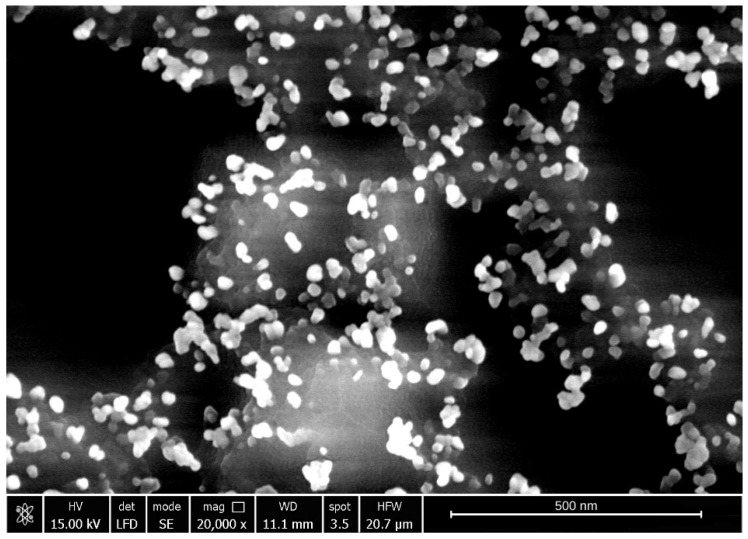
Scanning electron microscopy analysis of the sample synthesized under optimal conditions: Cu(II):TSC:starch = 1:2:0.2, pH 5, 85 °C, 45 min.

The surface morphology of the synthesized CuNPs was characterized using scanning electron microscopy (SEM), and a representative micrograph is depicted in Figure 9.

As observed in Figure 9, the CuNPs form nanocluster structures composed predominantly of spherical nanoparticles, exhibiting a relatively uniform spatial distribution. The predominance of a spherical morphology is particularly relevant for potential biological applications, as this geometry provides a high surface-to-volume ratio and facilitates predictable interactions with biological environments. Such characteristics are advantageous for applications including drug delivery systems, bioimaging, and antimicrobial strategies [7,41].

### 2.4. CuNPs in Biological Applications

#### 2.4.1. Impedance Based Real-Time Monitoring of Cell Viability

**Figure 10:**
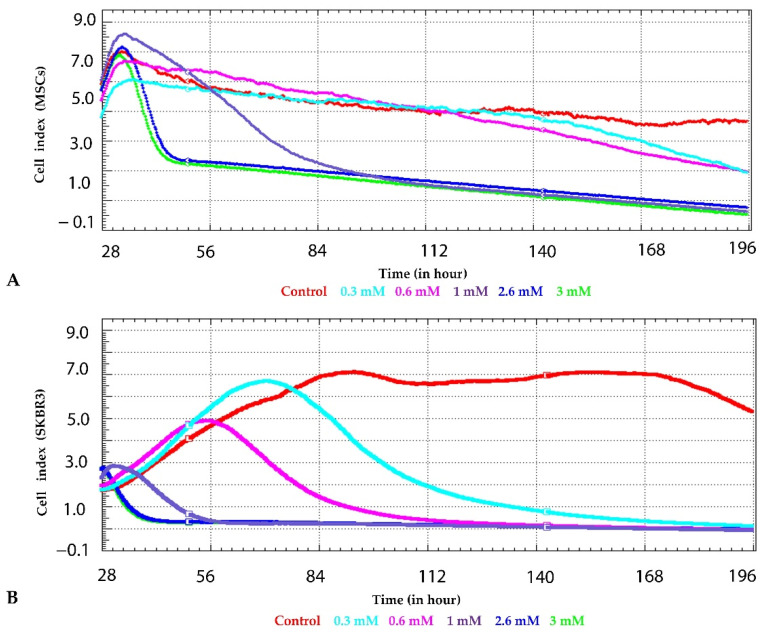
Dynamic impedance-based profiling of MSCs (Figure (**A**)) and SKBR3 cells (Figure (**B**)) carried out using xCELLigence platform. Cell responses were continuously recorded over a 7 day period following exposure to increasing concentrations of nanomaterial.

To investigate the impact of CuNPs on cell viability and proliferation capacity, continuous impedance-based measurements were carried out over a 196 h period using the xCELLigence (Santa Clara, CA, USA) real-time analysis platform. In cultured MSCs (Figure 10A), CuNP exposure induced rapid and concentration-dependent alterations in cell index dynamics. Shortly after the applied treatments (approximately 30–40 h), a transient increase in the cell index was observed at lower concentrations (0.3 mM, 0.6 mM), suggesting an initial adaptive response. This phase was followed by a progressive decline in cell index over time, indicative of impaired cell adhesion, and reduced proliferation, suggesting potential cytotoxic effects. Higher concentrations of 2.4 mM and 3 mM resulted in a more pronounced decrease in cell index, with values approaching baseline after prolonged exposure, consistent with sustained cytotoxicity. Overall, MSC cells displayed a time-dependent sensitivity to CuNPs, with delayed but persistent effects detectable through continuous real-time monitoring., In contrast, the breast cancer cell line SKBR3 exhibited a different response pattern to CuNP treatment (Figure 10B). In the first couple of hours following exposure, low-dose CuNPs showed an increase in cell index compared to control cells; however, the cell index collapsed in a dose-dependent manner. Higher concentrations of the synthesized nanoparticles, led to a rapid and substantial decrease in cell index, occurring earlier and being more pronounced than in MSCs. The experimental data indicate a well-controlled particle size and a highly uniform surface charge distribution for the synthesized copper nanoparticles (CuNPs).

An essential factor to take into consideration when evaluating the cytotoxic effects is the nanoparticle size. An increased cytotoxicity is associated with nanoparticles in the 40–60 nm size range, which appear to interact more strongly with cells, resulting in increased biological effects, whereas deviations toward smaller or larger dimensions may mitigate cytotoxicity [42]. The CuNPs used in this study had an average hydrodynamic diameter of 76.7 nm and displayed concentration-dependent cytotoxic effects, with SKBR3 being more susceptible compared to MSC.

#### 2.4.2. Annexin V–FITC/PI Flowcytometry Assay

**Figure 11:**
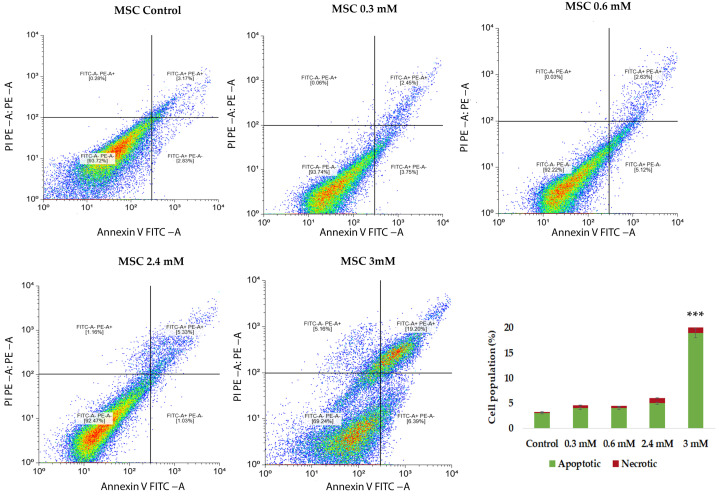
Flowcytometric analysis of MSCs exposed to increasing concentrations of copper content following Annexin V/PI staining. Viable cells are localized in the lower left quadrant (Annexin V^−^/PI^−^), early apoptotic cells in the lower right quadrant (Annexin V^+^/PI^−^), late apoptotic cells in the upper right quadrant (Annexin V^+^/PI^+^) and the necrotic or dead cells in the upper left quadrants (Annexin V^−^/PI ^+^). Data are presented as mean ± SD from three independent biological experiments (n = 3) across five experimental conditions. Statistical analysis was performed using one-way analysis of variance (ANOVA) followed by post hoc pairwise comparisons to determine differences between experimental groups. Statistical significance threshold: *** *p* < 0.001.

To further characterize the cytotoxic effect of CuNPs on MSCs, apoptosis and cell death were assessed using Annexin V–FITC/propidium iodide (PI) staining followed by flowcytometric evaluation. CuNP exposure induced a concentration-dependent increase in apoptotic cell population compared to the untreated control. At low concentrations of copper, the majority of MSCs remained viable, with a slight increase in the percentage of early apoptotic cells, indicating limited exposure of the membrane’s phosphatidyl serine and minimal cytotoxic stress (Figure 11). These findings are consistent with the observation from real-time measurements at low doses. However, higher concentrations of copper content resulted in a marked shift towards apoptotic and late apoptotic populations, accompanied by a small increase in necrotic cells. Overall, Annexin V–FITC/PI analysis confirms that CuNP-induced cytotoxicity in MSCs is predominantly mediated by apoptosis in a dose-dependent manner.

**Figure 12:**
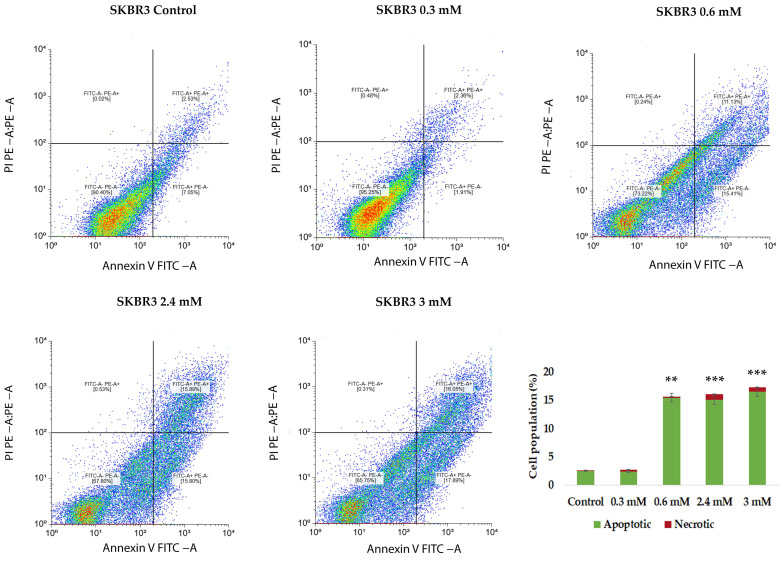
Flowcytometric analysis of SKBRs exposed to different concentrations of CuNPs following Annexin V/PI staining. Viable cells are presented in the lower left quadrant (Annexin V^−^/PI^−^), early apoptotic cells in the lower right quadrant (Annexin V^+^/PI^−^), late apoptotic cells in the upper right quadrant (Annexin V^+^/PI^+^) and the necrotic or dead cells in the upper left quadrants (Annexin V^−^/PI^+^). Data are presented as mean ± SD from three independent biological experiments (n = 3) across five experimental conditions. Statistical analysis was performed using one-way analysis of variance (ANOVA) followed by post hoc pairwise comparisons to determine differences between experimental groups. Statistical significance threshold: ** *p* < 0.01, *** *p* < 0.001.

A 24 h, CuNP treatment resulted in a clear concentration-dependent alteration of SKBR3 cell fate, characterized by a progressive reduction in the viable cell population and an increase in apoptotic cell number (Figure 12). At lower concentrations, SKBR3 cells exhibited a moderate increase in apoptotic cells, while the majority of the cells remained viable. At higher concentrations, a significant accumulation of late apoptotic cells was observed, accompanied by a marked decline in cell viability. A limited increase in PI-positive necrotic cells was also detected, suggesting that apoptosis represents the dominant mode of cell death, with necrosis contributing in small percentage only at higher nanoparticle doses. In line with previous reports on epithelial kidney and liver cell models, our results further support that CuNPs exert cytotoxic effects through oxidative stress-mediated dysfunction, leading to apoptosis; however, the cytotoxic effect appears to be strongly dependent on cell type and nanoparticle concentration [22].

**Figure 13:**
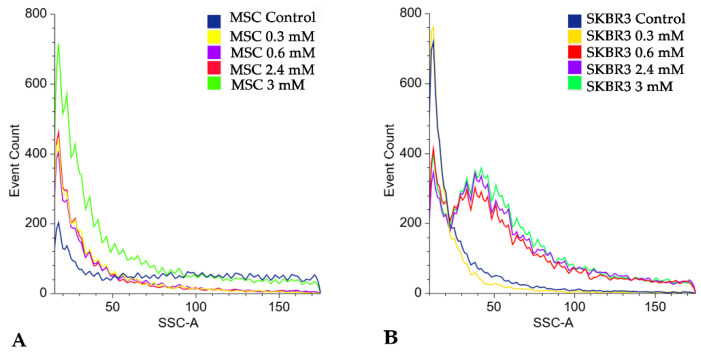
Flowcytometric analysis of the side scatter area (SSC-A) parameter indicating a dose-dependent increase in cellular granularity following CuNP treatment in MSC cells (**A**) and SKBR3 cells (**B**).

Side scatter area (SSC-A) analysis revealed a dose-dependent increase in cellular granularity following CuNP exposure, indicating a slightly enhanced intracellular complexity that might be associated with nanoparticle uptake and stress-induced structural alterations (Figure 13). In MSC cells the most pronounced increase was observed at the highest concentration (3 mM), whereas SKBR3 cells showed increased SSC-A values beginning at 0.6 mM and up to the highest concentration used of 3 mM, consistent with the progressive alterations observed in apoptotic markers.

#### 2.4.3. Assessment of Intracellular ROS by DCFH-DA Staining

**Figure 14:**
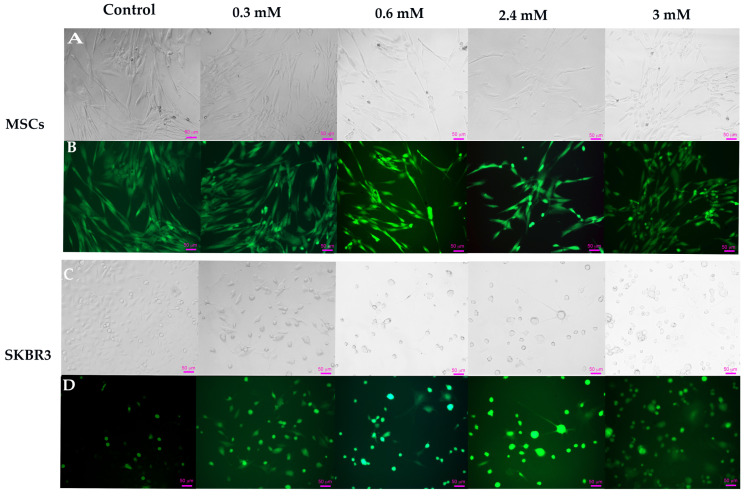
Representative phase-contrast and fluorescence microscopy images illustrating intracellular ROS generation following 2′7′-DCFH-DA staining. Bright fields images for MSC (**A**) and SKBR3 cells (**C**) along with their corresponding fluorescence images of MSC and SKBR3 cells (**B**,**D**). Scale bar: 50 µm.

Intracellular reactive oxygen species (ROS) generation was evaluated using 2′7′-dichlorodihydrofluorescein diacetate (DCFH-DA) 24 h after the CuNP treatments were applied. Following cellular uptake, DCFH-DA is deacetylated by intracellular esterase and subsequently oxidized by reactive oxygen species to the fluorescent compound 2′7′-dichlorofluorescein (DCF), enabling indirect quantification of oxidative stress [43]. Representative phase-contrast and fluorescence microscopy images illustrate CuNP-induced morphological alterations and intracellular ROS production in both cell types (Figure 14). In MSCs, control cells exhibited a typical fibroblast-like morphology and a higher basal ROS level, compared to control SKBR3 cells. The increased basal ROS level in MSC control cells is in line with previous research demonstrating the role of intracellular ROS in the regulation of mesenchymal stem cell fate and reflects a state of increased metabolic activity [43]. Upon CuNP exposure, intracellular ROS levels remained elevated in MSCs across all concentrations. At higher concentrations, 2.4 mM and 3 mM, pronounced morphological changes became evident in phase-contrast microscopy images, indicating a decrease in cell viability due to the cytotoxic effects associated with the higher copper concentration in the synthesized mixture.

In SKBR3 cells, control cells displayed low fluorescence intensity, associated with low basal intracellular ROS levels. Exposure to CuNPs, even at low concentrations, elicited a marked increase in fluorescence intensity, accompanied by early morphological changes such as cell rounding and reduced adhesion, indicating severe oxidative stress-induced damage and pronounced loss of cell viability. The increase in ROS following CuNP exposure intensifies the cellular redox burden, overwhelming the adaptive capacity of the cellular antioxidant system [44] and thereby promoting selective vulnerability of cancer cells compared to normal MSCs, which display greater resilience to CuNP-induced oxidative stress.

#### 2.4.4. Oxidative Stress-Related Gene Expression

**Figure 15:**
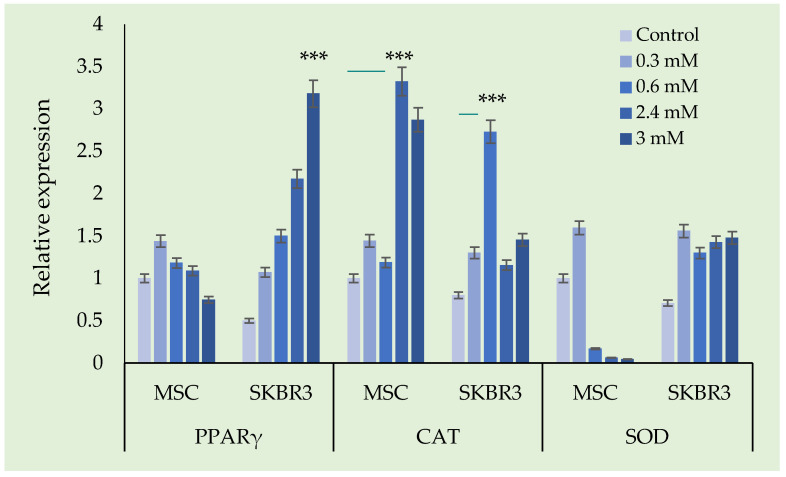
Changes in the expression of oxidative stress-related genes (PPARγ, SOD, and CAT) in response to CuNP exposure. GAPDH was used as the internal reference gene for normalization. Statistical significance threshold: *** *p* < 0.001. Visualization of qPCR amplification products for oxidative stress markers by agarose gel electrophoresis (PPARγ, SOD, CAT) are presented in Supplementary Files (Figure S1).

The relative gene expressions of PPARγ, CAT and SOD were evaluated in MSC and SKBR3 following exposure to increasing concentrations of the tested compound compared to untreated control. The results are presented in Figure 15.

In MSCs, PPARγ expression showed a modest increase at lower concentration, followed by a decrease at intermediate and higher doses. In contrast, SKBR3 cells exhibited a clear dose-dependent upregulation of PPARγ with a progressive increase in expression from 0.3 mM to 3 mM, (*p* < 0.0001).

CAT expression in MSCs increases slightly at lower concentrations, with the highest levels observed at 2.4 mM, followed by a small reduction at 3 mM. In SKBR3 cells, CAT expression was upregulated particularly at 0.6 mM, followed by a significant decline, though expression remained above control levels.

In MSCs, SOD expression increased slightly at 0.3 mM but was strongly suppressed at higher concentrations. SKBR3 cells showed an increase in SOD expression across all tested concentrations. The data presented here demonstrate that CuNPs induce distinct, cell-type specific alterations in the expression of PPARγ, CAT and SOD in MSCs and SKBR3 cells, suggesting differences in redox regulation and stress adaptation. Given the redox activity of CuNPs and their ability to generate ROS, the gene expression changes we observed reflect differential cellular strategies to manage oxidative stress. Oxidative stress is induced when cells experience an imbalance between prooxidant factors, such as an influx of CuNPs and the antioxidant defense system, leading to the accumulation of intracellular ROS. PPARγ functions like a key transcriptional regulator of the antioxidant response. Once activated, PPARγ directly modulates the expression of multiple redox-related genes, including CAT and MnSOD while suppressing the prooxidant enzymes. This integrated PPARγ–CAT–SOD axis represents an early adaptive response aimed at quenching excessive ROS, preserving mitochondrial function and promoting cell survival. However when the oxidative stress is sustained or exceeds the buffering capacity of the antioxidant system, this protective network becomes inefficient, resulting in irreversible cellular damage and activation of cell death pathways [45].

SKBR3 cells show a pronounced activation of the antioxidant pathway in response to CuNP exposure, as it was also observed after DCFH-DA staining. In contrast, MSCs, which display a higher basal level of ROS, showed an increase in PPARγ and CAT expression following CuNP treatment, while SOD increased only at the 0.6 mM concentration and was subsequently reduced at all the other concentrations tested.

Taken together, these findings suggest that CuNP-induced oxidative stress differentially engages PPARγ-mediated antioxidant pathways in cancer versus normal stem cells. While SKBR3 cells activate a robust yet ultimately insufficient antioxidant response that culminates in apoptotic cell death, MSCs display a greater capacity to withstand CuNP exposure, maintaining redox homeostasis without excessive activation of stress-induced apoptotic pathways.

## 3. Discussions

In our previous studies, CuNPs were synthesized using a conventional chemical reduction approach, which involved a strong reducing agent [12,46]. This strategy enabled the formation of small CuNPs (~37.5 nm) but the use of strong reducing agents is often associated with very fast reduction kinetics, limited control over particle growth, and potential drawbacks related to particle aggregation, residual chemical reactivity, and reduced environmental compatibility [47,48].

In our present work, a new method was developed using starch as both a mild reducing agent and as a stabilizer while trisodium citrate was added to control the quantity of Cu(II) ions in solution, thereby slowing the rate of reducing the metal and allowing further control of nanoparticle development. As a result, the optimal synthesis parameters were: Cu(II):TSC:starch = 1:2:0.2 M, pH = 5, homogenization time 45 min, and temperature 85 °C. The final CuNPs were uniform in size with an average hydrodynamic diameter of 76.7 nm and estimated physical diameter of around 85 nm, and they exhibited good colloidal stability as was shown through their very low zeta potential value (−57.4 mV), corresponding to an electrophoretic mobility of −4.47 × 10^−4^ cm^2^ V^−1^ s^−1^. The presence of a single narrow peak in the intensity-weighted size distribution confirms their monodisperse nature, while absolute zeta potential values exceeding ±30 mV indicate strong electrostatic repulsion that prevents agglomeration, supporting the formation of a highly stable colloidal system with minimal aggregation tendency. Compared to previously reported synthesized copper nanoparticles, usually over 80–100 nm (e.g., starch capped Cu_2_O nanocube’s ranging from 123 to 227 nm and plant extracts producing from 80 nm upwards), this method allows better control of particle size and dispersion under growth-favored conditions [43,44,45,46,47]. Nanoparticle size is a critical parameter influencing cytotoxicity, as cellular interactions, uptake and biological effects are dependent on particle size, with larger nanoparticles generally exhibiting reduced cytotoxic potential. One of the primary mechanisms underlying CuNP-induced cytotoxicity is ROS production. In response to oxidative imbalance, cells typically activate their antioxidant pathways, in order to neutralize ROS and limit cellular damage. However, when ROS accumulation exceeds the buffering capacity of the cell, oxidative-stress damage is installed disrupting the mitochondrial function, and ultimately leading to apoptotic cell death [48,49,50,51].

In our study the fluorescence microscopy analysis indicated a dose-dependent elevation of ROS in SKBR3 cells following CuNP exposure, correlating with the observed reduction in viability and increased apoptosis. These findings indicate that CuNP treatment drives oxidative stress in SKBR3 cells beyond the tolerance threshold, overwhelming the antioxidant defense systems of the cell. In contrast, normal MSCs exhibited greater resistance to CuNP-induced oxidative challenges, with significant cytotoxic and apoptotic effects detected only at the highest tested concentration. Notably, untreated MSCs, as primary cells, display relatively elevated basal ROS levels and a more efficient antioxidant defense system [43], which confers them a greater resilience towards oxidative CuNP-induced stress.

The biological activity of the presented CuNPs proved to be improved compared to the Cu-based nanomaterial previously synthesized via a chemical reduction method [46]. Using this new green synthesis approach, CuNPs exhibited a clear dose-dependent cytotoxic effect on SKBR3 cells at all five tested concentrations, although the percentages of apoptotic and necrotic cells were not markedly pronounced. This may be attributed to the stability and uniformity of the nanoparticles. In terms of oxidative stress, the present CuNPs showed a more controlled response than the smaller CuNPs due to their larger size and would induce apoptosis in cancer cells at a lower concentration than the previous formulation. A differential sensitivity between the normal cells and the cancer cells was observed, with MSCs displaying greater resilience and a much better-preserved morphology in culture following exposure to starch-mediated CuNPs. The use of starch in the formation of CuNPs, instead of using strong chemical reducing agents, may also be responsible for the development of nanoparticles that have better biocompatibility and fewer negative effects. Based on their mechanism of action, CuNPs appear to be particularly advantageous for targeting cancer cells, which often operate under pre-existing oxidative stress due to their higher metabolic rates, and therefore are more vulnerable to ROS amplification. The enhanced photothermal properties of CuNPs support their potential as dual therapeutic agents, particularly in breast cancer where laser-induced hyperthermia may amplify nanoparticle-mediated cytotoxicity [52].

Although SEM provided relevant information regarding particle shape, a limitation of the actual study is the absence of a very detailed morphological characterization of the synthesized CuNPs, this being a topic of further investigations. Nevertheless, for the biological applications, the hydrodynamic size distribution and colloidal stability in suspension that are measured by DLS and the zeta potential, are essential measurements. These parameters have a direct impact on nanoparticle–cell interaction, cellular uptake and potential cytotoxicity.

## 4. Materials and Methods

### 4.1. Synthesis of Copper Nanoparticles, CuNPs

To refine the synthesis of copper nanoparticles, a systematic evaluation of the main reaction parameters was carried out, namely the molar ratio between the CuNP precursor, reducing agent, stabilizing agent and complexing agent, as well as the influence of pH, temperature, and homogenization time. In this study, copper chloride dihydrate, CuCl_2_·2H_2_O 1M (Carl Roth, Karlsruhe, Germany) was used as the CuNP precursor and analytical starch (Sigma-Aldrich, Darmstadt, Germany) was used as the reducing and stabilizing agent. The complexing agent was trisodium citrate, TSC (Chimexim, Bucharest, Romania), Na_3_C_6_H_5_O_7_·3H_2_O 2M.

Initially, the trisodium citrate solution was first mixed with the copper precursor and magnetically stirred for 5 min to ensure adequate homogenization. Subsequently, a 0.2 M starch (pH = 13) solution was added to the mixture, being further homogenized for periods ranging between 15 and 60 min. This controlled approach allowed precise adjustment of nanoparticle properties, which is essential for tailoring CuNPs for specific applications.

To determine the optimal amount of complexing agent, the concentration of TSC was varied to achieve molar ratios of Cu(II):TSC:starch as 1:0.5:0.5, 1:1.5:0.5, 1:2:0.5 and 1:2.5:0.5.

For the measurement of the optimal amount of reducing agent, starch, the quantities of precursor and complexing agent were kept constant while varying the amount of starch to achieve molar ratios of Cu(II):TSC:starch equal to 1:2:0.1, 1:2:0.2, and 1:2:0.3.

To establish the optimal pH, the pH of the reaction mass was adjusted with a NaOH solution in the range of pH 5–11 using a Mettler Toledo pH meter (Mettler-Toledo International Inc., Columbus, OH, USA). The chosen Cu(II):TSC:starch ratio was the optimal ratio established previously.

Following the optimal reaction parameters, the optimum temperature was established by varying the temperature in the range of 25–85 °C.

### 4.2. Copper Nanoparticles Characterization

The synthesized CuNPs were characterized using comprehensive physicochemical techniques to evaluate their optical, structural, and chemical properties. UV–Vis spectroscopy was performed using a Varian Cary 50 spectrophotometer (Varian Inc., Santa Clara, CA, USA). Fourier transform infrared (FT-IR) spectroscopy was carried out using a Shimadzu IRAffinity-1S infrared spectrophotometer equipped with an ATR QATR-10 module (Shimadzu Corporation, Kyoto, Japan); additionally, a Bruker Platinum ATR-QL Diamond spectrometer (Bruker Corporation, Billerica, MA, USA) was employed for comparative measurements. The theoretical size of spherical CuNPs was estimated based on Mie theory. Particle size distribution and colloidal stability were assessed by zeta potential measurements using a HORIBA SZ-100 V2 nanoparticle analyzer (HORIBA Ltd., Kyoto, Japan). The structural morphology of the synthesized CuNPs was investigated by means of scanning electron microscopy (SEM) using a Quanta FEG 250 instrument (FEI Company, Hillsboro, OR, USA).

The copper concentration in the synthesized nanoparticles was determined to be 18.5 mg·L^−1^ by atomic absorption spectrophotometry using a Varian SpectrAAS 280 FS atomic absorption spectrophotometer (Varian Inc., Palo Alto, CA, USA). The corresponding Cu(II) concentrations (expressed in mM), were prepared by appropriate dilution of a 1 M CuCl_2_·2H_2_O stock solution. Subsequently, CuNP dispersions were diluted in order to obtain the final copper concentrations of 0.3, 0.6, 1.2, 2.4, and 3 mM, which were used in our in vitro experiments.

### 4.3. In Vitro Biological Evaluation

#### 4.3.1. Cytotoxicity Assessment

The effects of CuNPs were evaluated on normal MSCs and the SKBR3 breast cancer cell line. MSCs were isolated from dental pulp tissue following approved ethical guidelines, and the written informed consent was obtained from each subject. SKBR3 cells were purchased from the American Type Culture Collection (ATCC, HTB-30, Manassas, VA, USA). SKBR3, a well-characterized cell line, was selected to evaluate CuNPs as a representative in vitro model of HER 2 positive breast cancer cells, a clinically relevant and often aggressive subtype for which improved therapeutic strategies remain needed.

MSCs were cultivated in α-Minimum essential medium (α-MEM) (Pan-Biotech, Aidenbach, Germany) supplemented with 10% fetal bovine serum and 1% penicillin-streptomycin (Life Technologies, Grand Island, NY, USA). The SKBR3 cell line was cultivated in McCoy medium (Life Technologies, Paisley, UK), similarly supplemented. Cell proliferation and viability were monitored in real time using the xCELLigence system (Santa Clara, CA, USA), a label-free and non-invasive platform that measures changes in electrical impedance as an indicator of cell adhesion and viability. Cells were seeded at a density of 7 × 10^3^ cells per well, in complete media, and allowed to adhere overnight. The next day the cells were treated with the specified concentrations of CuNPs, while untreated cells served as controls. Cellular responses were recorded over seven days and analyzed using RTCA software (version 1.2.1, Santa Clara, CA, USA).

#### 4.3.2. Apoptosis Evaluation

The pro-apoptotic effects of CuNPs on normal MSCs and the SKBR3 breast cancer cell line were assessed using Annexin V–FITC/propidium iodide (PI) staining followed by flowcytometric analysis. Cells were seeded at 5 × 10^5^ cells per well, allowed to attach overnight, and then exposed to the CuNPs for 24 h in complete culture medium. This exposure duration was selected to induce measurable apoptotic responses while preserving a viable cell population for subsequent metabolic analyses. Following treatment, cells were collected, washed with phosphate-buffered saline, stained with FITC Annexin V Apoptosis Detection kit I (BD, Erembodegem, Belgium), according to the manufacturer’s protocol, and analyzed using a BD FACSAria III flow cytometer (Becton Dickinson Biosciences, Franklin Lakes, NJ, USA). For staining, 5 µL of Annexin V–FITC and 5 µL of propidium iodide were added, and the cells were incubated for 15 min at room temperature and in the dark. Subsequently 400 µL 1 × Binding buffer was added, and the cells were analyzed by flow cytometry. Apoptotic and necrotic populations were quantified using the latest version of Floreada.io software (latest version 3/30/25, https://floreada.io (accessed on 26 February 2026)).

#### 4.3.3. Assessment of Intracellular ROS by DCFH-DA

Intracellular reactive oxygen species (ROS) levels were evaluated by staining with 2,7 dichlorofluorescein diacetate (DCFH2-DA) (Sigma Aldrich, St. Louis, MO, USA). For this assay, 2 × 10^4^ cells/well were allowed to adhere overnight in IBIDI culture plates (IBIDI GmbH, Grafelfing, Germany). The following day, CuNPs were added and the cells were returned to the incubator for an additional 24 h. Subsequently, cells were incubated with 10 µM DCFH2-DA for 30 min, at 37 °C. Phase-contrast and fluorescent images were acquired using a Zeiss Axio Observer D1 phase-contrast microscope with fluorescence (Zeiss, Oberkoche, Germany).

#### 4.3.4. Oxidative Stress Analysis and Gene Expression Profiling (qPCR)

To assess changes in oxidative stress-related gene expression, cells were seeded at a density of 5 × 10^5^ cells per well and allowed to attach overnight prior to treatment. The following day, cells were exposed to variable concentrations of CuNPs, for 24 h in complete culture medium. Total RNA was then isolated using TRIzol reagent (Invitrogen, Carlsbad, CA, USA) according to the manufacturer’s instructions, and 1 µg of RNA was reverse transcribed into cDNA using the RevertAid First Strand cDNA Synthesis Kit (Thermo Scientific, Vilnius, Lithuania). Quantitative real-time PCR analysis was performed using SYBR green chemistry on a LightCycler 480 system (Roch, Basel, Switzerland), targeting key genes involved in oxidative stress regulation, including peroxisome proliferator-activated receptor gamma PPARγ, superoxide dismutase (SOD), and catalase (CAT). Primer sequences for quantitative real-time PCR (q-PCR) are presented in Table S1 (Supplementary Files).

#### 4.3.5. Statistical Analyses

Data were analyzed using one-way analysis of variance (ANOVA), followed by a post hoc pairwise *t*-test to determine the differences between experimental groups. Statistical analysis was performed using Microsoft Excel (Office Pro Plus, 2021). Parametric results are expressed as mean ± standard deviation (SD) from three independent experiments and values of *p* < 0.05 were considered statistically significant.

## 5. Conclusions

The present study demonstrates a sustainable and efficient “green” synthesis method for copper nanoparticles, replacing harsh reducing agents with starch. The optimization process identified the ideal parameters as a molar ratio of Cu(II):TSC:starch = 1:2:0.2, pH 5, and reaction temperature of 85 °C maintained for 45 min. These conditions are critical; the high temperature is necessary to induce the thermal swelling and bursting of starch granules, which releases glucose units that act as the primary reducing agents for Cu^2+^ ions. The use of starch as both a reductant and as a stabilizer resulted in highly stable nanoparticles.

The synthesized CuNPs were characterized by physicochemical analysis using UV–Vis spectroscopy, and at λ ~746 nm the presence of CuNPs was revealed. Also, the FT-IR spectroscopy characterization confirms that the CuNPs are effectively encapsulated by a hybrid-capping matrix of starch and citrate. The presence of O-H and C=O vibrational bands validates the successful adsorption of organic stabilizers. The correlation between the average hydrodynamic diameter (76.7 nm) and the high Zeta potential (57.4 mV) confirms the exceptional colloidal stability of the synthesized CuNPs. The higher negative zeta potential in DLS analysis indicated a strong repulsion force between the particles, which resulted in amplification or enhancement of stability. Furthermore, the close agreement between the experimentally determined hydrodynamic diameter and the theoretical particle size (~85 nm) obtained using MiePlot v4.6 highlights the monodispersity of the colloidal system and validates the optical modeling approach. Using SEM analyses, we confirmed the spherical morphology and a well dispersed distribution of the synthesized CuNPs. Together, these results demonstrate the successful production of stable, uniformly dispersed CuNPs, a key requirement for their reliable performance in biological and functional applications.

The present study demonstrates that the currently tested CuNP formulation induces intracellular ROS production in a dose specific manner and elicits distinct, cell-type specific responses in MSCs and SKBR3 cancer cells. These findings underscore fundamental differences in redox regulation and stress adaptation between normal and malignant cells. Cancer cells exhibit a higher susceptibility to ROS accumulation and cell death, predominantly through apoptotic mechanisms that could provide suitable targets for future biomedical and cancer therapy applications of the copper-based nanoparticles.

## Data Availability

Data is contained within the article and Supplementary Materials.

## Supplementary Materials

The following supporting information can be downloaded at: https://www.mdpi.com/article/10.3390/ijms27062559/s1.

## Abbreviations

The following abbreviations are used in this manuscript:

NPs	Nanoparticles
Cu-based NPs	Copper-based nanoparticles
MSCs	Mesenchymal stem cells
TSC	Trisodium citrate
DLS	Dynamic Light Scattering
FWHM	Full width half maxima
FT-IR	Fourier Transform Infrared Spectroscopy
LSPR	Localized surface plasmon resonance
FWHM	Theoretical full width at half maximum
FITC	Fluorescein isothiocyanate
PI	Propidium iodide
qPCR	Quantitative Polymerase Chain Reaction
PPARγ	Peroxisome proliferator-activated receptor gamma
CAT	Catalase
ROS	Reactive oxygen species
DCFH-DA	2′7′-dichlorodihydrofluorescein diacetate
DNA	Deoxyribonucleic acid
SHEDs	Stem cells from human exfoliated deciduous teeth
MEM	Minimum essential medium
cDNA	Complementary DNA
RNA	Ribonucleic acid
SOD	Superoxide dismutase
GAPDH	Glyceraldehyde-3-phosphate dehydrogenase
PPARs	Peroxisome proliferator-activated receptors
SD	Standard deviation
