# Supplementary material for: A Novel Green Synthesis Method of Copper Nanoparticles and Their Biological Effects on Cancer and Normal Cells
Source: Int J Mol Sci. 2026 Mar 11;27(6):2559. doi: 10.3390/ijms27062559 (PMC13027345; doi:10.3390/ijms27062559)
Supplement: Supplementary file 1 [file ijms-27-02559-s001.zip › Suplimentary files.pdf]

Supplementary materials

# A Novel Green Synthesis Method of Copper Nanoparticles and Their Biological Effects on Cancer and Normal Cells

**Maria-Alexandra Pricop[1,2,†], Adina Negrea[2], Ioan Bogdan Pascu[3,†], Mihaela Ciopec[2], Petru Negrea[2,] Iustina-Mirabela Cristea[1], Călin Adrian Tatu [1,4,*], Alexandra Ivan[1,4]**

[1] OncoGen Centre, County Hospital Pius Branzeu, 156 Liviu Rebreanu Blvd., RO-300736, Timisoara, Roma-nia; alexandra.pricop@oncogen.ro (M.A.P.); mirabela.cristea@oncogen.ro (I-M.C.);

[2] Department of Applied Chemistry and Environmental Engineering and Inorganic Compounds, Faculty of industrial Chemistry, Biotechnology and Environmental Engineering, Politehnica University Timisoara, Vasile Pârvan 6, 300223, Timisoara, Romania; adina.negrea@upt.ro (A.N.); mihaela.ciopec@upt.ro (M.C.); petru.negrea@upt.ro (P.N.);

[3] Renewable Energy Research Institute-ICER, Politehnica University of Timisoara, 138 Gavril Musicescu Street, 300501 Timisoara, Romania; ioan.pascup@upt.ro (I.B.P.)

[4] Department of Functional Sciences, Center of Immuno-Physiology (CIFBIOTEH), University of Medicine and Pharmacy "Victor Babes", Eftimie Murgu Sq. 2, Timisoara, 300041, Romania; ivan.alexandra@umft.ro (A.I.); tatu.calin@umft.ro (C.A.T.);

* Correspondence: tatu.calin@umft.ro (C.A.T.)

† These authors contributed equally to this work.

**Table S1.** Primer sequence for quantitative real-time PCR (q-PCR)

| Gene | Primer sequence |
|---|---|
| PPARγ | AAGACCACTCCCACTCCTTTG |
| | GTCAGCGGACTCTGGATTCA |
| SOD | GGTGGGCCAAAGGATGAAGAG |
| | CCACAAGCCAAACGACTTCC |
| CAT | TGGAGCTGGTAACCCATGAGG |
| | CCTTTGCCTTGGAGTATTTGGTA |

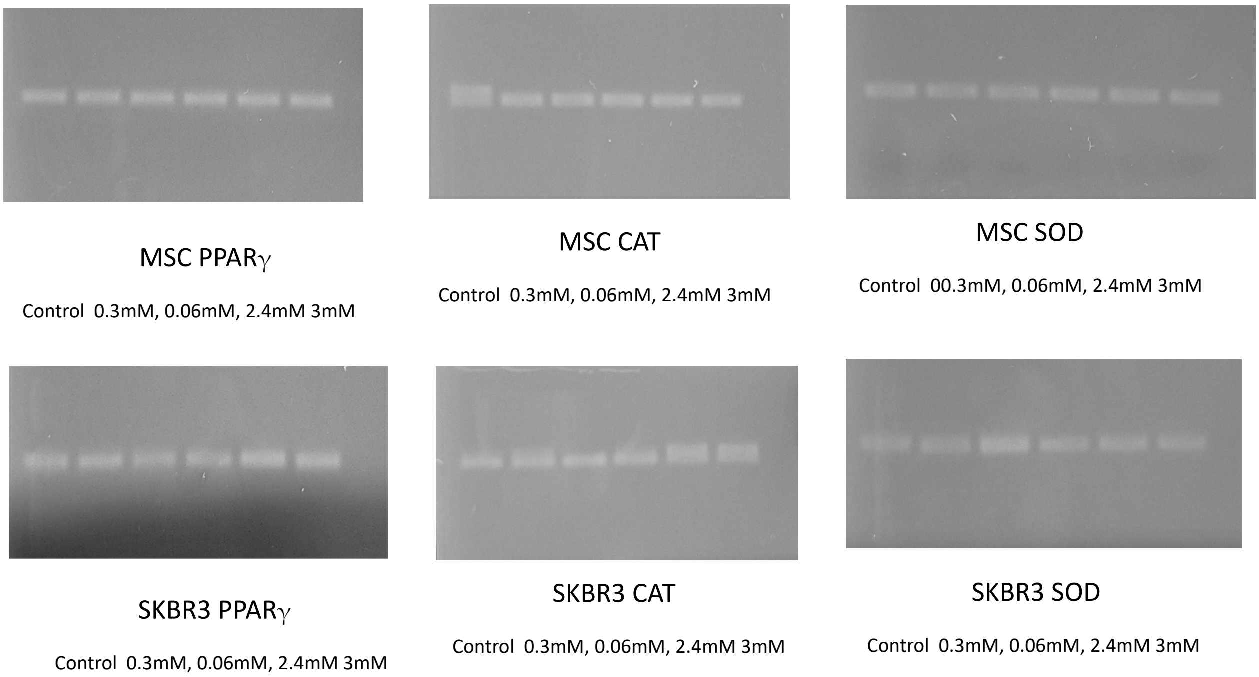


**Figure S1.** Visualization of qPCR amplification products for oxidative stress markers by agarose gel electrophoresis (PPARγ, SOD, CAT)
